# Clinical Outcome and Underlying Genetic Cause of Functional Terminal Complement Pathway Deficiencies in a Multicenter UK Cohort

**DOI:** 10.1007/s10875-022-01213-9

**Published:** 2022-01-27

**Authors:** Annalie Shears, Cathal Steele, Jamie Craig, Stephen Jolles, Sinisa Savic, Rosie Hague, Tanya Coulter, Richard Herriot, Peter D. Arkwright

**Affiliations:** 1 NIHR Paediatric Academic Clinical Fellow, University of Manchester, Manchester, UK; 2 NHS Tayside, Ninewells Hospital and Medical School, Dundee, Scotland; 3 University of Dundee, Dundee, Scotland; 4 University Hospital of Wales, Cardiff, Wales; 5 School of Medicine, University of Leeds, Leeds, & NIHR–BRC, Leeds, UK; 6 Immunology, Royal Hospital for Children, Glasgow, Scotland; 7 Immunology, Belfast Health and Social Care Trust, Belfast, Northern Ireland; 8 Immunology, Aberdeen Royal Infirmary, Aberdeen, Scotland; 9 Paediatric Immunology, Lydia Becker Institute, University of Manchester, Manchester, UK

**Keywords:** Terminal complement pathway, Factor H, Factor I, Meningococcal infection, Genetics

## Abstract

**Background:**

Terminal complement pathway deficiencies often present with severe and recurrent infections. There is a lack of good-quality data on these rare conditions. This study investigated the clinical outcome and genetic variation in a large UK multi-center cohort with primary and secondary terminal complement deficiencies.

**Methods:**

Clinicians from seven UK centers provided anonymised demographic, clinical, and laboratory data on patients with terminal complement deficiencies, which were collated and analysed.

**Results:**

Forty patients, median age 19 (range 3–62) years, were identified with terminal complement deficiencies. Ten (62%) of 16 patients with low serum C5 concentrations had underlying pathogenic *CFH* or *CFI* gene variants. Two-thirds were from consanguineous Asian families, and 80% had an affected family member. The median age of the first infection was 9 years. Forty-three percent suffered meningococcal serotype B and 43% serotype Y infections. Nine (22%) were treated in intensive care for meningococcal septicaemia. Two patients had died, one from intercurrent COVID-19. Twenty-one (52%) were asymptomatic and diagnosed based on family history. All but one patient had received booster meningococcal vaccines and 70% were taking prophylactic antibiotics.

**Discussion:**

The genetic etiology and clinical course of patients with primary and secondary terminal complement deficiency are variable. Patients with low antigenic C5 concentrations require genetic testing, as the low level may reflect consumption secondary to regulatory defects in the pathway. Screening of siblings is important. Only half of the patients develop septicaemia, but all should have a clear management plan.

**Supplementary Information:**

The online version contains supplementary material available at 10.1007/s10875-022-01213-9.

## Introduction

The complement system is an important component of innate immune defence against invading bacteria, particularly encapsulated meningococci and pneumococci. Terminal complement deficiencies are inherited as autosomal recessive traits and their prevalence varies, depending on consanguinity and family size [1]. The terminal complement pathway is made up of five components (C5–C9). They combine to form the pore-forming membrane attack complex (MAC) that disrupts membranes and lyse bacteria [2].

Deficiencies in terminal complement components impair immunity, particularly against meningococci [3]. Patients have a 7,000 to 10,000-fold higher risk of invasive meningococcal disease, and half have recurrent infections [4, 5]. Less prevalent meningococcal strains with lower virulence often cause invasive meningococcal infections in these patients [4, 6].

Good-quality data on this topic are sparse, with only a few published case reports or series [6–11]. The aim of this study was to characterise the clinical and laboratory features of a large UK cohort of patients with primary and secondary terminal complement deficiencies, to understand more fully the morbidity and mortality, thus providing information to guide future management decisions.

## Methods

### Patient Identification and Recruitment

This was a retrospective cohort study, in which clinicians working in major UK clinical immunology referral centers were emailed and invited to participate. Seven centers participated, with an overall response rate of 80%. Patients with primary or secondary terminal pathway complement immunodeficiencies defined by undetectable AP50 levels were identified from local databases at the referring clinical immunology center. Only patients where C5, C6, C7, C8, and C9 complement concentrations had been measured and a low level found were included. Ethics approval was obtained from the National UK Ethics Committee (IRAS number 259418).

### Data Collection and Analysis

Anonymised data containing no patient identifiers were entered into standardised Excel data sheets by the clinician with details of demographic, clinical, and laboratory parameters (CH50, AP50 and specific complement factors, genetic mutation screening, vaccine responses) and treatment received (vaccines, antibiotics, intensive care admissions).

Data were collated and analysed using IBM SPSS Statistics 25. Continuous variables are quoted as medians and ranges. For discrete variables, groups were compared using the chi-square test. Multivariate analysis was performed using binominal logistic regression. Differences were considered statistically significant if the *p* value was < 0.05.

### Complement Assays

Complement measurements were performed by accredited regional immunology laboratories using standard techniques [12]. Briefly, complement-dependent lysis of antibody-sensitised red blood cells in either a fluid or gel was used to provide a quantitative measure of functional activity (CH50 for the classical pathway and AP50 for the alternative pathway). Specific complement components of the alternate pathway and C5 to C9, or factor H and I were detected by either gel diffusion (Ouchterlony technique) or ELISA. Subsequent gene sequencing was initiated by physicians liaising with the UK accredited regional clinical genetics centers.

## Results

### Demographics

Forty patients with primary and secondary terminal complement deficiencies were identified from seven clinical immunology centers across the UK (Aberdeen, Belfast, Cardiff, Dundee, Glasgow, Leeds, Manchester) (Table 1, Table SI). Twenty-two (55%) patients were male. The median age at the last appointment or death was 19 (3–62) years. Ethnic backgrounds were white European (13, 32%) or Asian (27, 68%). Eighty percent had a family history of a terminal complement deficiency, and 26 (65%) had known consanguineous parenthood. All patients had undetectable AP50.

**Table 1:** Demographic characteristics of patients with terminal complement pathway deficiencies

	Total cohort	CF5	CF6	CF7	CF8	CFH	CFI
Number	40	6 (15%)	6 (15%)	9 (22.5%)	9 (22.5%)	2 (5%)	8 (20%)
Age at last review/death (years)	19 (3–62)	17 (3–20)	23 (16–62)	19 (9–40)	30 (6–60)	6 (3–8)	18 (7–30)
Age at diagnosis (years)	14 (1–45)	8 (1–16)	16 (12–30)	15 (1–45)	24 (8–27)	10 (7–14)	10 (3–16)
Gender (male)	22 (55%)	4 (67%)	3 (50%)	5 (56%)	5 (56%)	1 (50%)	4 (50%)
Ethnicity							
White European	13 (32%)	2 (33%)	2 (33%)	2 (22%)	5 (56%)	2 (100%)	-
Asian	27 (68%)	4 (67%)	4 (67%)	7 (78%)	4 (44%)	-	8 (100%)
Consanguinity	26 (65%)	4 (67%)	3 (50%)	7 (78%)	4 (44%)	-	8 (100%)
Family History	32 (80%)	5 (83%)	3 (50%)	9 (100%)	5 (56%)	2 (100%)	8 (100%)

Discrete variables, numbers (%); age, median (range)

**Fig. 1:**
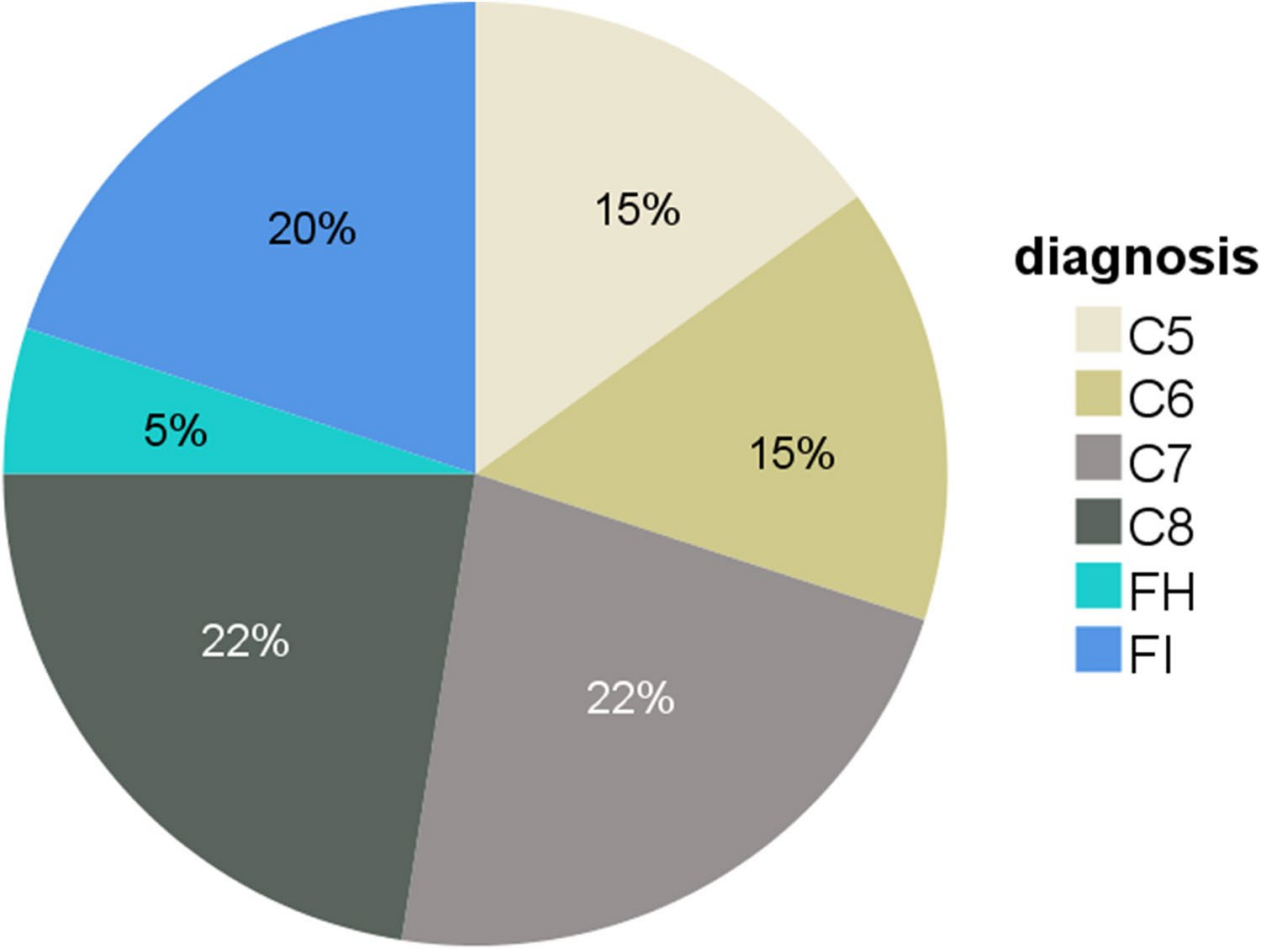
Distribution of primary and secondary terminal complement component deficiencies

Six (15%) patients were deficient in C5, six (15%) in C6, nine (22.5%) in C7, and nine (22.5%) in C8. Ten patients (25%) who had a low C5 concentration were subsequently found on genetic testing to have primary complement factor H or I, rather than C5 deficiency (Fig. 1).

### Clinical Infections

**Fig. 2:**
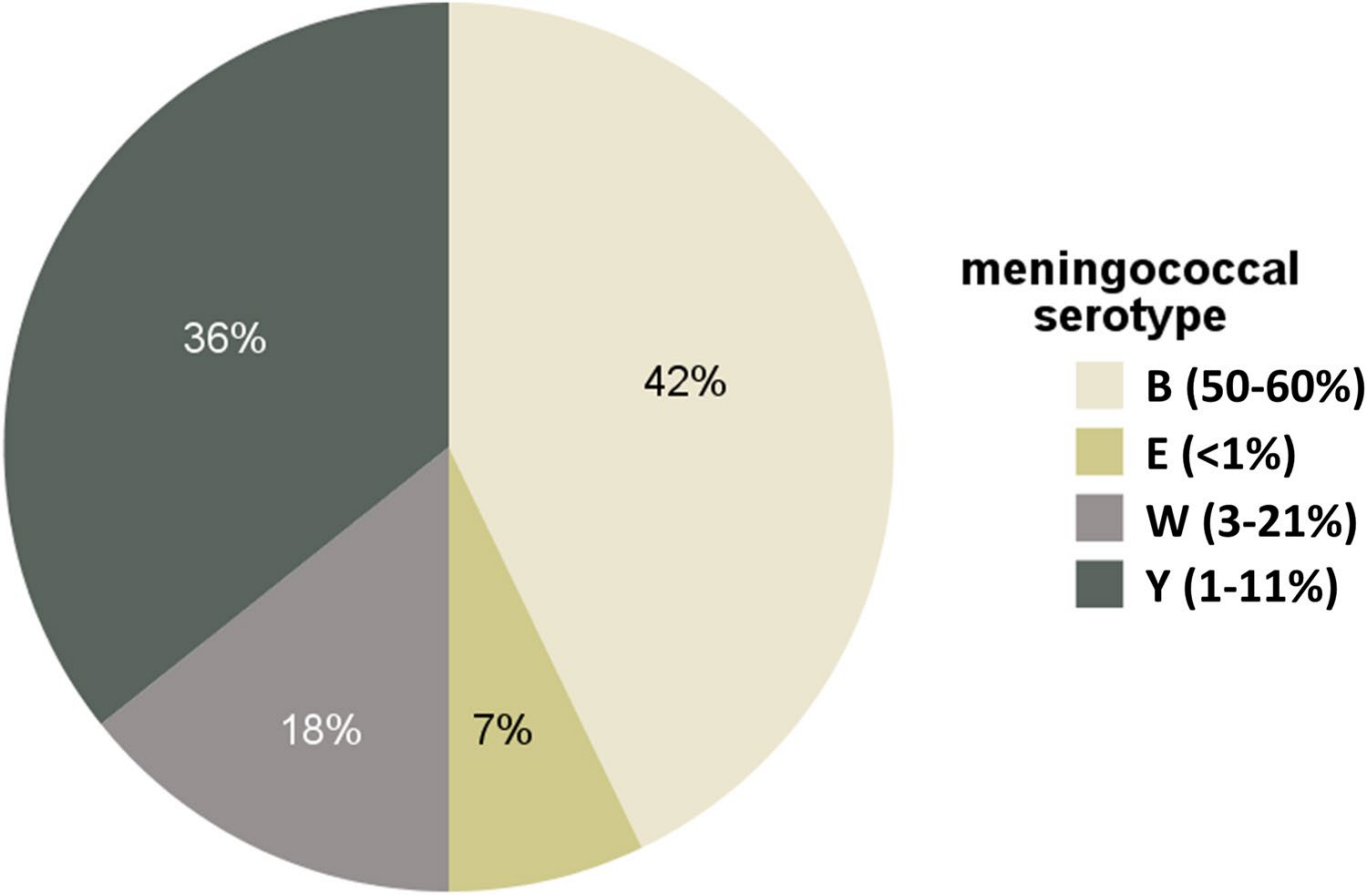
Meningococcal infection by serotype. The relative percentages of each serotypes associated with invasive meningococcal disease in England between 1999 and 2019 are quoted in brackets in the legend [13]

Nineteen (48%) patients had a history of meningococcal and/or other deep-seated bacterial infections. Eighteen (45%) patients suffered from meningococcal septicaemia, seven (18%) meningococcal meningitis, three (8%) meningococcal arthritis, and one (2%) meningococcal Fitz-Hugh Curtis syndrome. The median age at first meningococcal infection was 9 years (1–25 years), and the median number of infections was one (range 0–5) (Table 2). Of patients who developed meningococcal infections for which serotypes were identified (14 (67%) of 21), the majority were B (6, 43%) or Y (6, 43%). Serotypes W (1, 7%) and E (1, 7%) were also identified (Fig. 2). Non-meningococcal bacterial infections were observed in a third of patients, including upper and lower respiratory infections (7.5%), pyogenic meningitis (7.5%), osteomyelitis/septic arthritis (7.5%), and skin/soft tissue infections (10.0%) (Table SII). Two males (5%) died, one at 4 months old of pneumococcal meningitis, and another at 60 years old of COVID-19 not thought to be related to his complement deficiency.

**Table 2:** Meningococcal infections and mortality

	Total cohort 40	CF5 6 (15%)	CF6 6 (15%)	CF7 9 (22.5%)	CF8 9 (22.5%)	CFH 2 (5%)	CFI 8 (20%)
No. with no infection history	21 (52%)	4 (67%)	2 (33%)	7 (78%)	3 (33%)	0	5 (62%)
Age at first infection (years)	9 (1–25)	4 (0–10)	14 (4–15)	15 (8–16)	7 (1–25)	8 (1–14)	7 (1–10)
No. of meningococcal infections	1 (0–5)	0 (0–5)	2 (0–3)	0 (0–2)	2 (0–4)	2 (1–2)	0 (0–2)
Meningococcal serotypes B E W Y	14 6 (43%) 1 (7%) 1 (7%) 6 (43%)	4 2 (50%) - - 2 (50%)	4 2 (40%) - 1 (40%) 1 (20%)	3 1 (50%) - - 2 (50%)	1 - 1 (100%) - -	1 - - - 1 (100%)	1 1 (100%) - - -
Septicemia Meningitis Arthritis Fitz-Hugh Curtis syndrome	18 (45%) 7 (18%) 3 (8%) 1 (2%)	2 - - -	4 3 1 -	2 1 - -	5 3 1 1	2 - - -	3 - 1 -
Non-meningococcal sepsis	14 (34%)	2 (33%)	3 (50%)	1 (10%)	3 (33)	1 (50%)	4 (50%)
Deaths	2 (5%)	-	-	-	1	-	1

Discrete variables: numbers (%); age, median (range)

All patients without a family history of complement deficiency had suffered from meningococcal sepsis, while only 35% of patients with a family history developed sepsis (*p* < 0.001). Presentation with meningococcal sepsis was a major risk factor for admission to intensive care (relative risk 16.3, 95% confidence interval: 1.5 – 186). The risk of meningococcal septicaemia and admission to intensive care was not correlated with genetic diagnosis, age, gender, or antibiotic prophylaxis.

### Laboratory Findings

The results of genetic testing were available on 34 (85%) of the 40 patients (Table SI). Twenty-five (62.5%) had pathogenic homozygous gene variants, while in nine (22.5%) patients gene variants were compound heterozygous. Sixty-nine percent of patients with a history of consanguinity had a homozygous gene variant, compared with 36% of patients with no history (*p* < 0.05). Ten patients had antigenically low C5, but on genetic testing, eight had *CFI* gene mutations and two a *CFH* gene mutation indicating excessive C5 consumption rather than defective production.

### Management

Nine patients (22%) were admitted to the intensive care unit, and two patients were given FFP for fulminant meningococcaemia (Table 3). Ninety-eight percent of patients were given additional MenB/ MenACWY vaccines after diagnosis. Six patients (15%) also received an extra conjugate pneumococcal vaccine. Seventy percent of patients were prescribed either amoxicillin or penicillin prophylaxis, although 22% admitted to being noncompliant with antibiotic prophylaxis. The rate of compliance did not correlate with age, gender, ethnicity, intensive care admission, meningococcal septicemia, or meningitis.

**Table 3:** Clinical management

	Total cohort 40	CF5 6 (15%)	CF6 6 (15%)	CF7 9 (22.5%)	CF8 9 (22.5%)	CFH 2 (5%)	CFI 8 (20%)
Booster vaccinations MenB/ACWY PCV7/13 Hib	39 (98%) 6 (15%) 3 (8%)	6 1 1	6 - -	9 - -	9 1 1	2 - -	7 4 1
Vaccine responses checked	36 (90%)	6	3	9	9	2	7
Antibiotic prophylaxis Penicillin V Amoxicillin None Unknown Non-compliant	11 (28%) 17 (42%) 2 (5%) 1 (2%) 10 (22%)	1 4 - 1 1	4 - - - 2	- 7 - - 2	5 2 1 - 1	- 1 - - 1	1 3 1 - 3
Intensive care admission	9 (22%)	3	2	-	2	-	2
Fresh frozen plasma	2 (5%)	1	-	-	-	-	1

For discrete variables: numbers (%)

## Discussion

This is the largest retrospective, multicenter, cohort study of patients with terminal complement deficiencies. There are several lessons to be learned from the study.

Two-thirds of patients were from a consanguineous Asian family and 80% had an affected family member, highlighting the importance of testing siblings of index cases. The only patient to die as a direct complication of his complement deficiency (factor I) was a 4-month-old who succumbed to fulminant pneumococcal meningitis. The infant had three older siblings who were regularly being followed by the clinical immunology service. However, the immunology team was not made aware of the mother’s pregnancy nor the child’s birth. This tragic case illustrates the importance of ensuring early screening and a clear management plan for infants born to families with immune deficiency. A previous UK study screened 824 patients who had previously suffered from meningococcal sepsis for primary complement deficiency and found only one case [14]. Unlike their series, many of our cohorts had consanguineous parents and other affected siblings, highlighting the importance of taking a thorough family history in patients with meningococcal disease (especially with less common serotypes) or unusual or recurrent infections.

DNA was sent for genetic sequencing in the majority of patients, particularly those with low serum C5 concentrations. Ten (62%) of 16 patients with low C5 antigenemia had *CFH* or *CFI* pathogenic gene variants with normal *C5* genetics, consistent with a diagnosis of secondary consumptive C5 deficiency due to a primary defect in the regulatory complement component. We recommend that all patients with a suspected complement deficiency should have confirmatory genetic testing. Complement factors H and I regulate the formation of C3 and C5 convertase enzymes and cause excessive consumption [15]. In contrast, to complete deficiencies of complement factors suffered by patients in our cohort, haplo-insufficiencies of these regulatory factors are often associated with normal serum protein levels and overactivity of the complement pathway, leading to atypical haemolytic uremic syndrome or other vasculopathies [16, 17]. In addition, those patients treated with anti-C5 biologics such as eculizumab or ravulizumab also have a higher risk of meningococcaemia [18]. No C9 deficient patients were detected in this series, in keeping with the absence of this immune deficiency in the UK and other European Primary Immunodeficiency registries [19]. In contrast, C9 deficiency has been documented in some Japanese patients with terminal complement deficiency [20].

This is one of the first studies to document the proportion of family members with terminal complement deficiency who are asymptomatic. Fifty-two percent of patients (median age of 17 (range 3–46) years) had no prior history of bacterial sepsis. Asymptomatic adults cannot be excluded based on the absence of clinical disease and their complement pathway should be tested. It is also important to be aware that the median age of first meningococcal infection in these patients was 9 (range 1–25) years, while in the UK, the incidence of meningococcal infections is highest in infants under one year old [21]. In keeping with our findings, Platonov et al. previously found that the mean age of first meningococcal infection in their cohort of terminal complement deficient patients was 15 years old [22]. Thus, there should be a higher index of suspicion of complement defects in patients presenting later in childhood or beyond.

Patients with defects in terminal complement deficiencies typically suffer from meningococcal sepsis as was the case with nearly half of the patients in this cohort [23]. The relatively low mortality per episode of meningococcal disease in patients with terminal complement deficiencies has been demonstrated previously, hypothesised as due to lower endotoxin release from the bacterial surface in the absence of a complete terminal complement pathway [22]. Deep-seated non-meningococcal infections are also common, occurring in one-third of patients, and include bacterial meningitis, osteomyelitis/septic arthritis, sinopulmonary infection, and soft-tissue infections.

Conjugate meningococcal B and ACYW and pneumococcal and *Haemophilus influenzae* type B vaccines should be given to all patients with complement deficiencies, with subsequent monitoring of antibody titres. Prophylactic antibiotics are recommended, particularly in patients with recurrent meningococcal infections or those at higher risk of endemic or occupational exposure [24]. The use of prophylactic antibiotics should be balanced against the potential development of resistance. Patients also need education, including an emergency plan in the event of infection and travel. Some patients have emergency antibiotics at home and MedicAlert or similar bracelets. The effectiveness of these measures in preventing disease is not possible to determine from the present study. This study found no association between either rate of infection or admission to intensive and receiving antibiotic prophylaxis but did not formally assess adherence. Twenty-two percent of patients admitted not to take their antibiotic prophylaxis, but there were no obvious predictive demographic or clinical correlates. Adherence and factors influencing it are generic issues that would be possibly better addressed in studies of adherence to medications given for more common conditions, e.g., asthma, hypertension, and HIV.

In summary, this study highlights the complexities of terminal complement deficiencies. Meningococcal infection over 5 years of age and with less common serotypes (e.g. E, Y) or recurrent meningococcal infection should prompt further investigation of terminal pathway defects, particularly where there is a family history of unusual or deep-seated bacterial infections or consanguinity. The importance of genetic testing for possible factor H and I deficiency in patients with low antigenic C5 levels is also emphasised. Further studies investigating the prevalence of terminal complement deficiencies and the impact of antibiotic prophylaxis and vaccination on outcome are required. The use of international databases may enable long-term follow-up of these patients.

## Supplementary Information

Below is the link to the electronic supplementary material.

Supplementary file1 (DOCX 31 KB)

## Data Availability

Data is available on request to PDA, the corresponding author.

## Abbreviations

CFH: Complement factor H
CHI: Complement factor I
MAC: Membrane attack complex
