# Supplementary material for: Clinical Outcome and Underlying Genetic Cause of Functional Terminal Complement Pathway Deficiencies in a Multicenter UK Cohort
Source: J Clin Immunol. 2022 Jan 27;42(3):665–71. doi: 10.1007/s10875-022-01213-9 (PMC8793329; doi:10.1007/s10875-022-01213-9)
Supplement: Supplementary file 1 — Supplementary file1 (DOCX 31 KB) [file 10875_2022_1213_MOESM1_ESM.docx]

**SUPPLEMENTARY DATA**

**Clinical outcome and underlying genetic cause of functional terminal complement pathway deficiencies in a multi-centre UK cohort**

Annalie Shears^1^, Cathal Steele^2^, Jamie Craig^3^, Stephen Jolles^4^, Sinisa Savic^5^, Rosie Hague^6^, Tanya Coulter^7^, Richard Herriot^8^, Peter D. Arkwright^9^

**Table SI Clinical and laboratory feature of individual patients**

| **Patient** | **age** | **sex** | **consan** | **FH** | **alive** | **age**  **1st inf** | **age**  **Dx** | **No.**  **inf** | **disease** | **prophylaxis** | **CH50** | **AP50** | **component** | **gene variant** |
| --- | --- | --- | --- | --- | --- | --- | --- | --- | --- | --- | --- | --- | --- | --- |
| 1 | 19 | m | yes | yes | yes |  | 14 | 0 | none | A | U/D | U/D | C5 <22.2mg/L  (90-172) | C5 c.1561C>A/  c.1924_1925delAG |
| 2 | 15 | m | yes | yes | yes | 6 | 7 | 2 | S | A | U/D | U/D | C5 <22.2mg/L  (90-172) | C5 c.1561C>A/  c.1924_1925delAG |
| 3 | 12 | m | yes | yes | yes |  | 10 | 0 | none | A | U/D | U/D | C5 <22.2mg/L  (90-172) | C5 c.4426C>T/  c.4426C>T |
| 4 | 3 | m | yes | yes | yes | 1 | 1 | 0 | M (Pn) | A | U/D | U/D | C5 <22.2mg/L  (90-172) | C5 c.4426C>T/  c.4426C>T |
| 5 | 19 | f | no | yes | yes | 1 | 2 | 0 | Ar (Pn) | N/C | U/D | U/D | C5 <22.2mg/L  (90-172) | C5 c.4426C>T/  c.4426C>T |
| 6 | 20 | f | no | no | yes | 10 | 16 | 5 | S | N/C | 18U/ml  (23-46) | U/D | C5 <22.2mg/L  (90-172) | C5 c.68dupA/  c.4426C>T |
| 7 | 17 | m | yes | yes | yes | 13 | 13 | 2 | S | P | U/D | U/D | C6 <4.2mg/L  (45-96) | C6 c.1759C>T/  c.1759C>T |
| 8 | 16 | m | yes | yes | yes |  | 12 | 0 | none | P | U/D | U/D | C6 <4.2mg/L  (45-96) | C6 c.1759C>T/  c.1759C>T |
| 9 | 20 | m | no | no | yes | 4 | 16 | 3 | S M Ar | N/C | U/D | U/D | C6 <4.2mg/L  (45-96) | C6 c.821delA/  c.143G>A |
| 10 | 35 | f | yes | yes | yes |  | 21 | 0 | none | N/C | U/D | U/D | C6 <4.2mg/L  (45-96) | C6 exon 7 deletion/  C6 exon 7 deletion |
| 11 | 26 | f | no | no | yes | 14 | 15 | 2 | S M | P | U/D | U/D | C6 <4.2mg/L  (45-96) | C6 exon 7 deletion/  C6 exon 7 deletion |
| 12 | 62 | f | no | no | yes | 15 | 30 | 3 | S M | P | U/D | U/D | C6 <4.2mg/L  (45-96) | N/A |
| 13 | 9 | f | yes | yes | yes | 8 | 9 | 1 | S | A | U/D | U/D | C7 <5.9mg/L  (55-85) | C7 c.1561C>A/  c.1924_1925delAG |
| 14 | 15 | f | yes | yes | yes |  | 14 | 0 | none | A | U/D | U/D | C7 <5.9mg/L  (55-85) | C7 c.1561C>A/  c.1924_1925delAG |
| 15 | 14 | f | yes | yes | yes |  | 13 | 0 | none | A | U/D | U/D | C7 <5.9mg/L  (55-85) | C7 c.1561C>A/  c.1924_1925delAG |
| 16 | 17 | m | yes | yes | yes |  | 16 | 0 | none | A | U/D | U/D | C7 <5.9mg/L  (55-85) | C7 c.1561C>A/  c.1924_1925delAG |
| 17 | 42 | m | no | yes | yes | 16 | 22 | 2 | M | N/C | U/D | U/D | C7 <5.9mg/L  (55-85) | C7 c.63-1G>A/  c.63-1G>A |
| 18 | 19 | m | yes | yes | yes |  | 18 | 0 | none | A | U/D | U/D | C7 <5.9mg/L  (55-85) | C7 c.1924_1925delAG/  c.1924_1925delAG |
| 19 | 46 | f | yes | yes | yes |  | 45 | 0 | none | A | U/D | U/D | C7 <5.9mg/L  (55-85) | C7 c.1924_1925delAG/  c.1924_1925delAG |
| 20 | 32 | m | yes | yes | yes |  | 1 | 0 | none | A | U/D | U/D | C7 <5.9mg/L  (55-85) | N/A |
| 21 | 33 | m | no | yes | yes | 15 | 15 | 1 | O | N/C | U/D | U/D | C7 <5.9mg/L  (55-85) | N/A |
| 22 | 11 | m | yes | yes | yes | 7 | 8 | 0 | Ar (Sa) | P | U/D | U/D | C8 <7.0mg/L  (40-80) | C8A c.630C>A/  c.630C>A |
| 23 | 15 | f | yes | yes | yes | 15 | 15 | 1 | A | A | U/D | U/D | C8 <7.0mg/L  (40-80) | C8A c.630C>A/  c.630C>A |
| 24 | 6 | m | yes | yes | yes |  | 14 | 0 | none | A | U/D | U/D | C8 <7.0mg/L  (40-80) | C8A c.630C>A/  c.630C>A |
| 25 | 40 | m | no | no | yes | 4 | 26 | 4 | none | P | U/D | U/D | C8 <7.0mg/L  (40-80) | C8B c.271C>T/  c.1282C>T |
| 26 | 30 | f | no | no | yes | 25 | 25 | 1 | S | N/C | 11U/ml  (23-46) | U/D | C8 31.0mg/L  (40-80) | C8B c.1282C>T/  c.1282C>T |
| 27 | 30 | m | yes | no | yes |  | 22 | 2 | S M | - | U/D | U/D | C8 <7.0mg/L  (40-80) | C8B c.150dupC/  c.150dupC |
| 28 | 60 | m | no | no | died  COVID 2020 |  | 24 | 4 | O | P | U/D | U/D | C8 <7.0mg/L  (40-80) | N/A |
| 29 | 46 | f | no | yes | yes | 1 | 27 | 2 | S M O | P | U/D | U/D | C8 <7.0mg/L  (40-80) | N/A |
| 30 | 42 | f | no | yes | yes |  | 25 | 0 | none | P | U/D | U/D | C8 <7.0mg/L  (40-80) | N/A |
| 31 | 8 | m | no | yes | yes | 1 | 7 | 1 | S O | A | U/D | U/D | C5 <22.5mg/L,  C3 <0.04mg/L (0.8-1.6) | CFH c.2T>C/  c.2T>C |
| 32 | 3 | f | no | yes | yes | 14 | 14 | 2 | S O | N/C | U/D | U/D | C5 <22.5mg/L,  C3 <0.04mg/L (0.8-1.6) | CFH c.2T>C/  c.2T>C |
| 33 | 14 | f | yes | yes | yes |  | 10 | 0 | none | A | U/D | U/D | FI <2.4mg/L, C5 <22.5mg/L,  C3 <0.04mg/L | CFI c.764G>A/  c.764G>A |
| 34 | 13 | f | yes | yes | yes | 3 | 3 | 1 | S M  (Pn) O | P | U/D | U/D | FI <2.4mg/L, C5 31.5mg/L,  C3 0.4mg/L (0.6-1.6) | CFI c.764G>A/  c.764G>A |
| 35 | 7 | m | yes | yes | yes | 1 | 7 | 0 | M (Pn) O | A | U/D | U/D | FI <2.4mg/L, C5 31.5mg/L,  C3 0.4mg/L (0.6-1.6) | CFI c.764G>A/  c.764G>A |
| 36 | 17 | m | yes | yes | died  M 2014 |  |  | 0 | M (Pn) | - | U/D | U/D | FI <2.4mg/L, C5 <22.5mg/L,  C3 0.2mg/L (0.8-1.6) | CFI c.1440_1441delAG/  c.1440_1441delAG |
| 37 | 30 | f | yes | yes | yes | 10 | 11 | 2 | S O | A | U/D | 8U/ml  (25-45) | FI <2.4mg/L, C5 <22.5mg/L,  C3 0.2mg/L (0.8-1.6) | CFI c.1440_1441delAG/  c.1440_1441delAG |
| 38 | 19 | m | yes | yes | yes |  | 5 | 0 | none | N/C | U/D | 7U/ml  (25-45) | FI <2.4mg/L, C5 <22.5mg/L,  C3 0.4mg/L (0.8-1.6) | CFI c.1440_1441delAG/  c.1440_1441delAG |
| 39 | 27 | m | yes | yes | yes |  | 14 | 0 | none | N/C | U/D | 7U/ml  (25-45) | FI <2.4mg/L, C5 <22.5mg/L,  C3 0.4mg/L (0.8-1.6) | CFI c.1440_1441delAG/  c.1440_1441delAG |
| 40 | 29 | f | yes | yes | yes | 10 | 16 | 2 | S Ar O | N/C | U/D | 212U/ml  (392-1019) | FI <2.4mg/L, C5 <22.5mg/L,  C3 0.4mg/L (0.8-1.6) | CFI c.1440_1441delAG/  c.1440_1441delAG |

age: years, cons: consanguinity, FH: family history, inf: infection, Sex: m: male, f: female, M: Diseases: S: septicemia, M: meningitis, Ar: arthritis, O: other (tonsillitis, pneumonia, otitis, sinusitis), Pn: pneumococcal (NB if not specified meningococcal), Sa: salmonella, Prophylaxis: A: amoxycillin, P: penicillin V, N/C: non-compliant, CH50/AP50: U/D: below detectable assay range, N/A not available

**Table SII Non-meningococcal infections**

| Infection | Number (%) |
| --- | --- |
| **Respiratory**   - Pneumococcal pneumonia | **3 (7.5%)**  1 (2.5%) |
| - Otitis media | 1 (2.5%) |
| **Meningitis (Pneumococcal)** | **3 (7.5%)** |
| **Osteomyelitis / septic arthritis (1 Pneumococcal, 1 Salmonella)** | **3 (7.5%)** |
| **Skin / subcutaneous tissues** | **4 (10.0%)** |
| - Cellulitis | 3 (7.5%) |
| - Skin abscess | 1 (2.5%) |
| **TOTAL** | **13 (32.5%)** |
